# Multiple spindle cell hemangiomas in both lungs: a rare case report and review of the literature

**DOI:** 10.1186/s13019-019-0906-y

**Published:** 2019-05-02

**Authors:** Xiao Duqing, Wu Zhaohong, Wang Gefei

**Affiliations:** Department of Thoracic Surgery, The Third Affiliated Hospital of Guangzhou Medical University, Guanzhou, Guandong China

**Keywords:** Spindle cell hemangiomas, Hemangioma of lung, Multiple nodules of lung

## Abstract

**Background:**

Spindle cell hemangioma (SCH) was an extremely rare benign tumor which typically arised in the subcutis of the distal extremities of young people. In this study, we reported a case of multiple spindle cell hemangioma in both lungs.

**Case presentation:**

A 19-year-old HIV-negative female was found to have multiple lung nodules by the chest X-ray during the physical examination. Her chest CT scan revealed multiple round-like pulmonary nodules in both lungs. Based on the morphological features and immunohistochemical examination for vascular markers CD31, CD34 and D2–40, the mass was diagnosed as SCH after surgery.

**Conclusion:**

SCH was an extremely rare tumor especially in both lungs. It should be considered in differential diagnosis of multiple lung nodules. Pathological features, the expression of CD31, CD34 and D2–40 could help to diagnosis of SCH.

## Background

Spindle cell hemangiomas which occur in both lungs were rare and they were not discussed fully in the published literature. Spindle cell hemangioma commonly arose in the subcutis, especially at the distal extremities. It frequently happened under the skin or under the mucous membrane. Herein we presented a case in which spindle cell hemangiomas were discovered on the lungs incidentally.

## Case presentation

A 19-year-old HIV- negative female was found to have multiple lung nodules by the chest X-ray during the physical examination. She had no symptoms at all such as chest tightness, cough or low fever. Physical examination and routine laboratory data showed no other abnormalities. Tumor markers (CEA, AFP, CA19–9, CYFRA21-1, NSE and SCC) were all negative. Sputum was negative for acid-fast bacilli in three occasions.

**Fig. 1:**
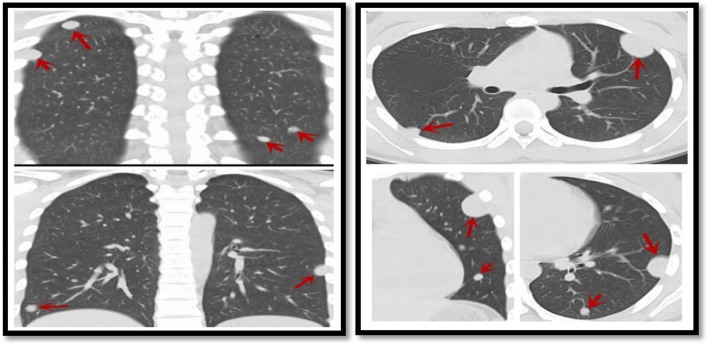
Chest CT (Multiple spindle cell hemangiomas in both lungs)

**Fig. 2:**
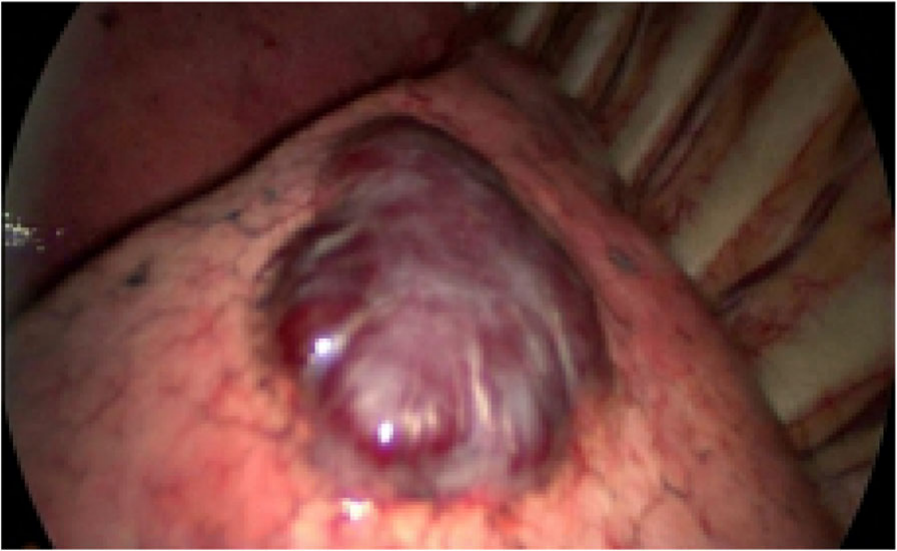
Operative findings

The chest computer tomography (CT) revealed multiple round nodules in both lungs. Nodules have smooth borders and the density of the nodules is relative uniform. The largest one is located near the apex of the upper lobe of the left lung measuring 2.2 × 2.4 × 2.1 cm in size by CT (Fig. 1). We initially believed that these lesions are either primary benign tumor such as leiomyomas or reactive processes like tuberculosis since clinical and radiographic findings did not suggest any malignancy. A diagnostic biopsy was performed in the left thoracic cavity through thoracoscopy. In the course of the procedure, many dark-red masses which had different size were identified in the left lung (Fig. 2). A tumor with a size of about 2.0 × 3.0 cm was wedged.

**Fig. 3:**
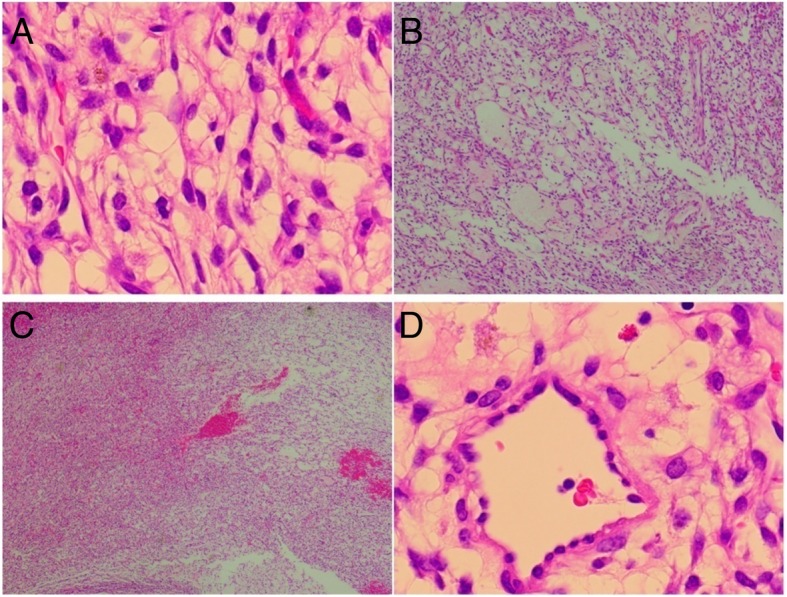
Histopathological features showing spindle cell proliferation. **a**. (40×), **b**. (20×), **c**. (20×), **d**. (40×))

**Fig. 4:**
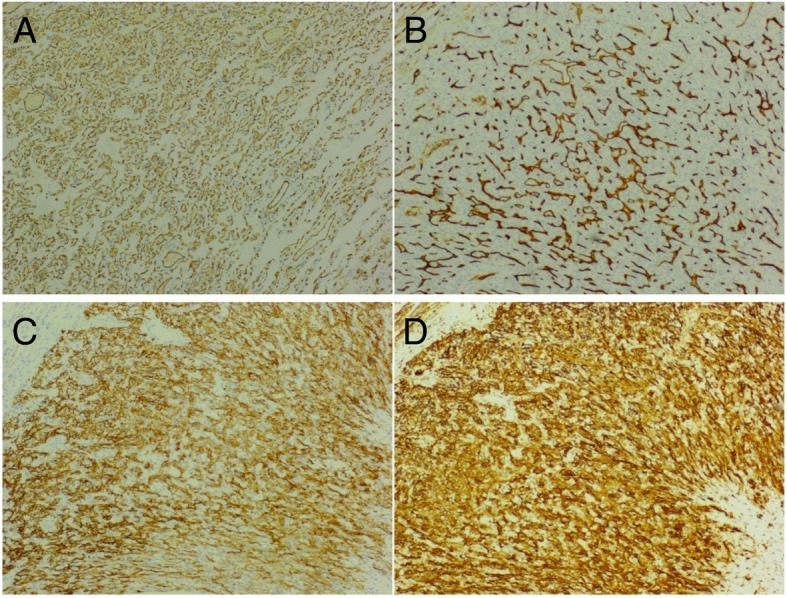
Immunohistochemical (**a**. CD31(+), **b**. CD34(+), **c**. CD56(−), **d**. D2–40(+))

**Fig. 5:**
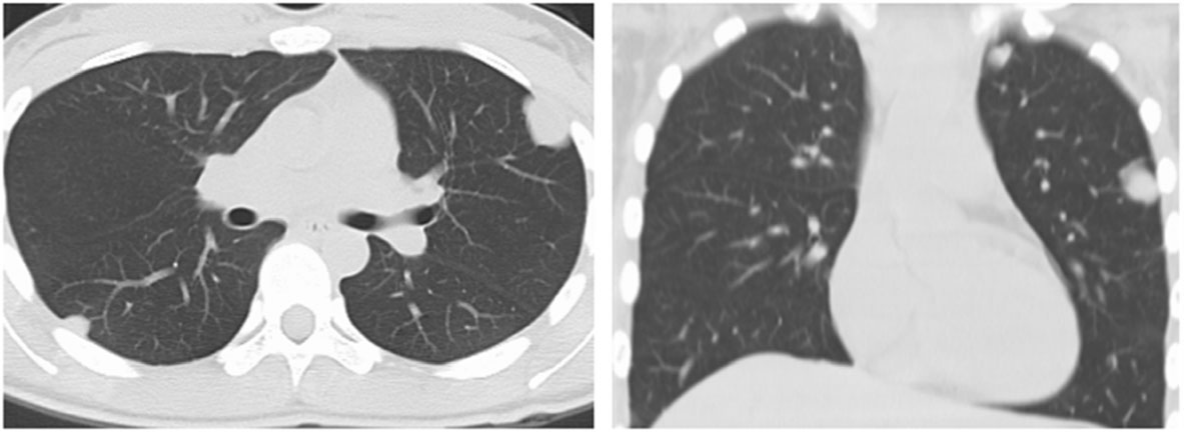
Chest CT (15 Apr. 2019)

Microscopically, the tumor was composed of thin-walled vessels lined by flattened endothelial cells. Stromal cells between vascular spaces were spindled or round, some of which are vacuolated. The nuclei ware medium-sized and mitotic figures were rare (Fig. 3). Immunohistochemical stains for vascular markers CD31, CD34 and D2–40 were positive and SMA was also positive in this tumor. HHV-8 was negative (Fig. 4). Morphological features were those of pulmonary spindle cell hemangioma. On follow-up, 15 months after surgery, the patient was asymptomatic, and did not show any signs of tumor growth through the chest CT in 15 Apr. 2019 (Fig. 5).

## Discussion

In 1986, Weiss and Enzinger described a new variant of vascular tumor designated spindle cell hemangioendothelioma which was considered has a limited malignant potential [1]. Later, the entity was defined as spindle cell hemangioma based on the analysis of a larger series, owing to the lack of metastasis, although local recurrence may occur [2]. Spindle cell hemangioma was a neoplasm that most commonly arises in the subcutis at the distal extremities. It usually presented under the skin or under the mucous membrane. There were some case-reports that SCH occured in muscles, retroperitoneum, mediastinum and even spinal cord [3]. So far, the case of multiple spindle cell hemangiomas in both lungs has not been reported yet in the English literature.

Spindle cell hemangioma had a similar incidence in men or women, mostly presents as red-purple nodules under the skin. Otherwise, the spindle cell hemangioma was growth slowly, and the size of nodules was usually 1~2 cm [1, 2]. In our case, the chest CT features were multiple, calcification occasionally, well-demarcated, homogeneous mass, which was difficult to distinguish with several benign and malignant lung tumors. CT and MRI were useful to determine the location of tumor and guide the feasibility of operation [3, 4]. Preoperative diagnosis of spindle cell hemangioma was difficult, it mainly relied on postoperative pathological analysis and immunohistochemistry [4].

The major differential diagnoses of multiple nodules in both lungs included pulmonary metastases, tuberculosis, lymphoma, sarcoidosis, and fungal infection. Other diseases which happened rarely in clinic included rheumatoid nodules and pulmonary amyloidosis. Since tuberculosis was not uncommon disease in China, the first thing was to rule out TB. The patient had no fever, no history of tuberculosis and no intimate relationship with tuberculosis patients recently. The sputum smear was negative for acid-fast bacilli and T-spot was also negative. Therefore, tuberculosis was less likely. Secondly, malignancy should be ruled out. However, the patient had no smoking history, no other site malignancy and no tumor markers (CEA, AFP, CA19–9, CYFRA21-1, NSE and SCC) positive. To make a definite diagnosis, wedge biopsy was performed. Postoperative pathological analysis showed characteristic features of spindle cell hemangioma. Wang L. [5] reported that, in our case, the endothelial cells in spindle cell hemangioma were positive for CD31 and Prox1, focally positive for D2–40. The major differential diagnosis of spindle cell hemangioma included Kaposi sarcoma, Kaposi-like hemangioendothelioma, cavernous hemangioma and epithelioid hemangioendothelioma. But, whether the morphological or pathological features, there was no evidence that it is the disease such as above.

Radiographically, this pulmonary spindle cell hemangioma showed features of smooth border, homogenous density and no infiltrated pattern. Pulmonary epithelioid hemangioendothelioma (PEH) should always be excluded. PEH was a rare vascular neoplasm that usually occurs in the lung and liver and often presented as bilateral multiple lung nodules. PEH often showed multifocal areas of reticulonodular patterns [6, 7]. Histologically, they were different and spindle cell hemangioma showed vascular tumor with spindle cell stroma without epithelioid appearance shown in PEH. Although immuonhistochemical stains could lead to a diagnosis of vascular origin, they were no able to differentiate these two entities. Morphological features did not fit into Kaposi sarcoma with negative HHV-8 in this HIV negative woman. Kaposi sarcoma was unlikely, Kaposi-like hemangioendothelioma usually occurred in much younger individuals.

As we searched the literature, no pulmonary spindle cell hemangioma had been reported yet. Management and prognosis of pulmonary spindle cell hemangioma had no consensus yet. It is considered as a benign vascular neoplasm. Observation and surgical resection are in consideration.

## Conclusion

SCH is an extremely rare tumor especially in both lungs. It should be considered in differential diagnosis of multiple lung nodules. Pathological features, the expression of CD31, CD34 and D2–40 can help to diagnosis of SCH.

## Abbreviations

CEA: Carcinoembryonic antigen; NSE: Neuron-specific enolase
CYFRA21-1: Cytokeratin-19fragments; PEH: Pulmonaryepithelioid emangioendothelioma
SCC: Squamous cell carcinoma antigen; AFP: alphafetoprotein
SCH: Spindle cell hemangioma; CT: Computer tomography

## References

[1] Weiss SW, Enzinger FM (1986). Spindle cell hemangioendothelioma. A low-grade angiosarcoma resembling a cavernous hemangioma and Kaposi's sarcoma. Am J Surg Pathol.

[2] Perkins P, Weiss SW (1996). Spindle cell hemangioendothelioma. An analysis of 78 cases with reassessment of its pathogenesis and biologic behavior. Am J Surg Pathol.

[3] Hakozaki M (2012). Intraosseous spindle cell hemangioma of the calcaneus: a case report and review of the literature. Ann Diagn Pathol.

[4] Marušić Z, Billings SD (2017). Histopathology of spindle cell vascular tumors. Surg Pathol Clin.

[5] Wang L, Gao T, Wang G (2014). Expression of Prox1, D2-40, and WT1 in spindle cell hemangioma. J Cutan Pathol.

[6] Woo JH, Kim TJ, Lee KS, KIM BT (2016). Epithelioid hemangioendothelioma in the thorax: Clinicopathologic,CT, PET, and prognostic features. Medicine (Baltimore).

[7] Sardaro A, Bardoscia L, Petruzzelli MF (2014). Epithelioid hemangioendothelioma:an overview and update on a rare vascular tumor. Oncol Review.

